# Evaluation of the Effect of Ciprofloxacin and Vancomycin on Mechanical Properties of PMMA Cement; a Preliminary Study on Molecular Weight

**DOI:** 10.1038/s41598-020-60970-y

**Published:** 2020-03-04

**Authors:** Marzieh Gandomkarzadeh, Hamid Reza Moghimi, Arash Mahboubi

**Affiliations:** 1 Department of Pharmaceutics and Nanotechnology, School of Pharmacy, Shahid Beheshti University of Medical Sciences, Tehran, Iran; 2 Food Safety Research Center, Shahid Beheshti University of Medical Sciences, Tehran, Iran

**Keywords:** Drug discovery, Engineering, Materials science

## Abstract

Antibiotic-loaded bone cement (ALBC) is commonly used in joint replacement therapy for prevention and treatment of bone infection and mechanical properties of the cement is still an important issue. The effects of ciprofloxacin and vancomycin was investigated on mechanical characterization of PMMA bone cement. Different properties of cement containing (0, 2.5, 5 and 10% W/W) antibiotics, including compressive and bending properties, microstructural, porosity and density were evaluated. Both antibiotics significantly reduced the density values and mechanical properties (compressive and flexural strength and modulus) in all groups in comparison to control over first two weeks (p < 0.05). This reduction was due to increased porosity upon antibiotic addition (3.05 and 3.67% for ciprofloxacin and vancomycin, respectively) in comparison to control (2.08%) (p < 0.001) and exposure to aqueous medium. Vancomycin as antibiotic with higher molecular weight (MW = 1485) had significant effect on compressive strength reduction of the cement at high amount compared to ciprofloxacin (MW = 367) (P < 0.01), there was no difference between two antibiotics at lower concentrations (P > 0.05). The effect of antibiotic loading is both molecular weight and drug content dependent. The time is also an important parameter and the second week is the probably optimum time to study mechanical behavior of ALBC.

## Introduction

Antibiotic-loaded bone cement (ALBC) has been provided successful and cost-effective prevention and treatment of orthopedic infections for many years^1,2^. The concept of bone cement containing antibiotics as a delivery vehicle was first introduced by Buchholz and Engelbrecht in 1970’s to prevent and treat musculoskeletal infections. It allows the local release of high concentrations of antibiotics without the complications and toxicity of systemic drug administration. Nowadays, low-dose antibiotic-impregnated bone cement (1 g antibiotic(s) in 40 g PMMA) is used as prophylaxis in orthopedic surgery. However, cements with higher content of antibiotics (usually 4 g antibiotics/40 g PMMA) are recommended in the treatment of orthopedic infections^3–5^. Many antibiotics including ciprofloxacin and vancomycin are widely used in this field. Ciprofloxacin can treat infections which have been caused by *Pseudomonas* and *Staphylococcus*. Vancomycin can also reduce the number of microorganisms in prosthesis or bone infections, and it is the choice treatment in methicillin-resistant *Staphylococcus aureus* (MRSA) infections^6,7^.

Addition of antibiotics may affect the mechanical properties of acrylic bone cement despite the wide clinical use of them in this drug delivery system^8^. In addition to cement properties, concentration and physicochemical properties of antibiotic like molecular weight can also affect the mechanical behavior of PMMA cement^9–12^.

To the best of our knowledge there is no study comparing the effects of ciprofloxacin and vancomycin addition in bone cement. These drugs have similar water solubility but different molecular weights. The purpose of this study was to investigate mechanical properties of cement impregnated with these antibiotics by means of static mechanical tests over 28 days.

## Materials and Methods

### Materials

Commercial acrylic bone cement, FIX 1 Radiopaque cement containing powder and liquid component was purchased from Groupe lépine (Genay, France) (Table 1). FIX 1 cement has medium/ standard viscosity that been used for digital application. This cement with a controlled setting time (7 ± 1 min) reaches to maximum temperature (57 ± 0.5 °C) for adapted polymerization. Amorphous Vancomycin HCl (vancomycin) with average particle size of 104.90 ± 9.14 μm was obtained from Livzon Pharmaceutical Group (Zhuhai, China). Crystalline ciprofloxacin HCl (ciprofloxacin) with average particle size of 42.49 ± 5.36 μm was obtained from Temad (Mashhad, Iran).

**Table 1:** Composition of FIX 1 Radiopaque cement.

Component	wt.%
Liquid (14.4 g)	
Methyl methacrylate (MMA) (monomer)	85.3
Butylmethacrylate	13.2
N,N-Dimethyl-p-toluidene	1.5
Hydroquinone	20 ppm
Powder (40 g)	
Polymethyl-methacrylate	87.6
Benzoyl peroxide	2.4
Barium sulphate	10

### Preparation of acrylic bone cement specimens

The *in vitro* study has been performed on acrylic bone cement specimens (small cylinders and rectangular strips) prepared by manual addition of antibiotics to bone cement. Different groups containing different concentrations of each antibiotic (2.5, 5 and 10% W/W) and control group were prepared. The required amount of the antibiotics and bone cement powder were mixed homogenously^13^. After that, the cement’s liquid monomer was added to the powder mixture, at a powder to liquid ratio of 2.77:1, in a glass deep dish. Mixing was carried out under a fume hood at room temperature (23 ± 1 °C) to achieve a state of doughy according to commercial BC manufactures instructions and ISO5833 (Implants for surgery—Acrylic resin cements) recommendations for 1 min^14,15^. After remaining the mixture cement for 90 sec, it was introduced into the rectangular strip molds (75 × 10 × 3.3 mm) to prepare the specimens for the bending test, and into small cylindrical molds (6 × 12 mm) for the compression test, accordingly with the ISO5833 mold dimensions. The final weight of prepared specimens for bending and compression tests was 3.20 and 0.42 g, respectively (Table S1). Bone cements were approximately 30 min pressurized between two plates and held until the cement had fully polymerized. Rough specimen edges were treated by sanding with 400 grade emery paper^16^.

### Biomechanical tests

The effects of antibiotics on the static mechanical properties of PMMA bone cements were evaluated by studying compressive and flexural strength and flexural and compressive modulus after adding 0% (as control) and 2.5 to 10% of the antibiotics. According to ISO 5833, cement measurement and running the tests (time zero) should be started at 24 ± 2 h after mixing of the cement^14^. To simulate the ageing process, about 400 specimens were immersed individually in 10- and 100-ml distilled water container at 37 ± 1 °C for compression and bending tests, respectively, and the fluid replaced completely on days 1, 7, 14, 21, and 28. The samples were taken out of incubator and dried^12,17–20^. The control samples stored in dry conditions/ room temperature (23 ± 1 °C) acted as absolute control for the control samples immersed in distilled water. The tests were performed at room temperature in a universal testing machine; model H25KS (Hounsfield Test Equipment Ltd., Redhill, UK) as described below^14^. Testing was carried out on five samples of each concentration. According to ISO 5833, the minimum requirement for average compressive and bending strength and bending modulus is 70, 50 and 1800 MPa, respectively^14^.

The compression test was carried out on cylindrical specimens according to ISO5833. The load-displacement curve was achieved using a constant crosshead speed, 20 mm/min, until reaching the upper yield point. The compressive strength (S_C_) and the compressive modulus (E_C_) were calculated by Eqs. (1) and (2):

1 $${S}_{C}=F/A$$

2 $${E}_{C}=\Delta \delta /\Delta \varepsilon $$

Where F is the upper yield-point load, A is cross section area of test specimen, $\Delta \delta$ is $\Delta {F}_{i}/A$, as *F*_*i*_ is the applied load at the point i of the linear section of the curve, $\Delta \varepsilon$ is $\Delta {l}_{i}/L$, where L is the length of the sample, and ${l}_{i}$ is the displacement corresponding to load *F*_*i*_ at a point in the linear section of the curve^18^.

The three-point bending tests were performed on rectangular strip specimens of the bone cement with different concentrations of the antibiotics. The distance between the two rods used in bending test in the lower part (span) was 60 mm and the gaps outside the rods were equal. The distance between the upper rod and lower rods was also equal. Rate of crosshead motion was calculated according to ASTM D790-03^18,21^. The test machine operated to produce load-deflection curve until the sample breaks. The flexural strength (S_F_) and the flexural modulus (E_F_) were calculated in accordance with Eqs. (3) and (4):

3 $${S}_{F}=3FL/2w{t}^{2}$$

4 $${E}_{F}={L}^{3}m/4w{t}^{3}$$

Where F is the force at break, L is the support span, w is the width of specimen, t is the thickness of specimen, and m is the slope of the tangent to the linear section of the curve^18,21^.

### Solid-state characterization

The intact and fracture surfaces of rectangular strip specimens, including cement without antibiotic as the control and cement which is containing 10% of two antibiotics were studied using a scanning electron microscope, SEM, model Philips XL 30 (SEM Tech Solutions, Billerica, USA). Both of dry and immersed samples have been evaluated for SEM analysis. Briefly, 3 mm samples of a selected group of fractured samples were used. The surfaces of samples were coated with a gold layer using a sputter coater model SCD 005 (BAL-TEC, New York, USA) under vacuum^22,23^. The specimen surfaces were scanned with SEM at an accelerating voltage of 25 kV with different magnifications. One specimen from each group was selected for the SEM analysis.

The porosity of the cement samples was determined using mercury intrusion porosimetry Pascal 140 (CE Elantech, Lakewood, USA) through the cylindrical cement. While the intrusion volume was recorded, the pressure was gradually increased to 200 MPa. As pressure is applied, mercury fills the larger pores first. As pressure increases, the filling proceeds to smaller and smaller pores. Total cumulative volume, total porosity, average pore diameter and bulk density were measured in this method^24^.

### Statistical analysis

All experiments were carried out five times and reported as mean ± SD. Biomechanical test results were analyzed by one-way analysis of variance (ANOVA) followed by LSD post-hoc analysis using IBM SPSS 21 software for Windows (Statistical Package for Social Science, IBM SPSS, New York, USA). P-value less than 0.05 was considered significant. Additionally, two-tailed Spearman correlation analysis was performed to study the relationship between cement strength and drug concentration.

## Results

### Biomechanical tests

Table 2 shows the comparison of biomechanical test for dry samples of control bone cement after 24 h storage in room temperature (23 ± 1 °C) and distilled water (37 ± 1 °C). After aging, strength for cement was found to be considerably lower than in the absolute control group. Immersion of cement in distilled water resulted in 7.50 and 13.15% reduction of compressive and flexural strength in control group, respectively.

**Table 2:** Compression and bending properties of control bone cements after one day storage in room temperature, 23 ± 1 °C (Dry sample) and distilled water, 37 ± 1 °C (Wet sample) (Mean (SD), n = 5).

Properties	Compressive strength (MPa)	Compressive modulus (GPa)	Flexural strength (MPa)	Flexural modulus (GPa)
Dry sample	89.88 (0.9)	1.72 (0.05)	57.33 (3.24)	2.65 (0.04)
Wet sample	83.21 (1.04)	1.61 (0.06)	49.79 (4.47)	2.35 (0.04)
Reduction (%)	7.50	6.04	13.15	11.32

**Figure 1:**
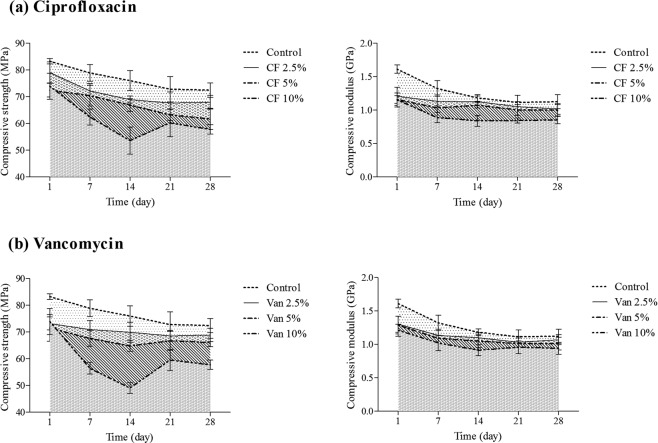
The effect of different concentrations of ciprofloxacin (CF) and vancomycin (Van) (0% ----; 2.5% ─; 5% -─-, 10%--─) on the compressive properties of bone cement after ageing in distilled water at 37 ± 1 °C over 28 days (Mean ± SD, n = 5).

The compressive strength in presence of 2.5, 5 and 10% of ciprofloxacin after the first day of study were significantly decreased to 5, 13 and 14%, respectively, indicating partially linear relation to the drug content (Fig. 1a, r^2^ = 0.838, P < 0.001). There was also a significant difference between control group and vancomycin- loaded cement for compressive strength (ANOVA, P < 0.01). Impregnation of 2.5, 5 and 10% of this drug led to decreases equal to 7, 15 and 35.5%, respectively, on day 14. These results showed a linear correlation between the reduction of compressive strength and the concentration of vancomycin up to 10% (Fig. 1b, r^2^ = 0.935, P < 0.001). The cement containing 2.5% of both antibiotics showed acceptable compressive strength according to ISO5833 standard level (<70 MPa) over 28 days, while addition of 5 and 10% of the drug led to unacceptable results^14^ (Fig. 1).

**Figure 2:**
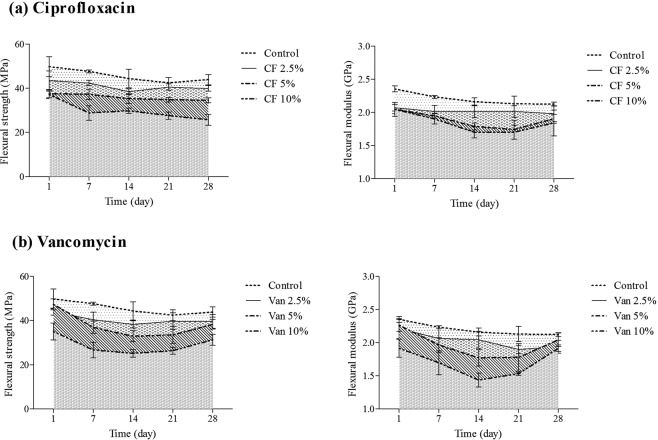
Comparison of the effect of different concentrations of ciprofloxacin (CF) and vancomycin (Van) (0% ----; 2.5% ─; 5% -─-, 10%--─) on the bending properties of bone cement after ageing in distilled water at 37 ± 1 °C over 28 days (Mean ± SD, n = 5).

The compressive strength of the cement containing 2.5% ciprofloxacin and vancomycin, decreased up to 8 and 7% on day 14 and 3 and 4% on day 28, respectively (Fig. 1). The flexural strength reduction in the cement containing 2.5% ciprofloxacin and vancomycin were equal to 13 and 13.5% on day 14 and 9 and 9.5% on day 28, respectively (Fig. 2). Since the properties of all groups including control group decreased in the first 14 days and increased in days 21 and 28 (Figs. 1 and 2), the immersion time was also an effective factor in the both of compressive and flexural properties of the cement.

The compressive and flexural modulus also showed similar results. These parameters were also drug concentration dependent and decreased in first two weeks and increased over time (Figs. 1 and 2). Flexural modulus reduction in cements containing 2.5, 5 and 10% of ciprofloxacin were equal to 6.5, 17 and 21%, respectively, on day 14 and were equal to 6.5, 10 and 14%, after 28 days (Fig. 2a). Flexural modulus of the cement containing 2.5, 5 and 10% vancomycin decreased to 5, 18 and 33%, respectively, on day 14, while these values increased on day 28 (Fig. 2b).

**Figure 3:**
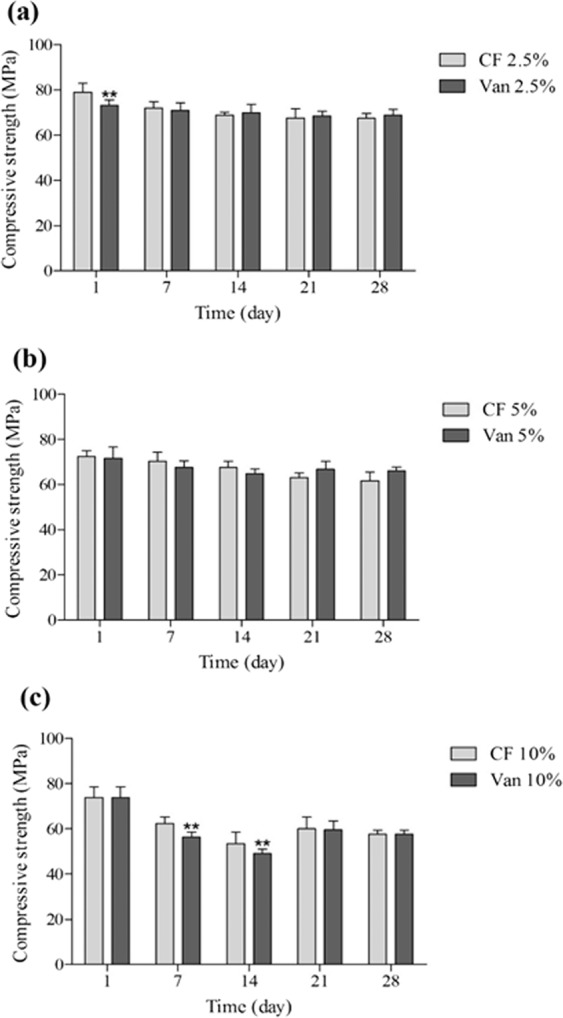
The compression strength of bone cements containing 2.5, 5 and 10% of ciprofloxacin (CF) and vancomycin (Van) for 28 days (MPa, Mean ± SD, n = 5), **P < 0.01; statistically different from ciprofloxacin group.

Comparison of the effect of two drugs showed the molecular weight of the drug could affect the cement strength at high concentration. There was significant difference between reduction of compressive strength after addition of 10% ciprofloxacin (25.8%) and vancomycin (35.5%) on day 14 (Fig. 3c, P < 0.01), while there was no significant difference at the concentrations of 2.5 and 5% (Fig. 3a,b, P > 0.05).

### Solid-state characterization

**Figure 4:**
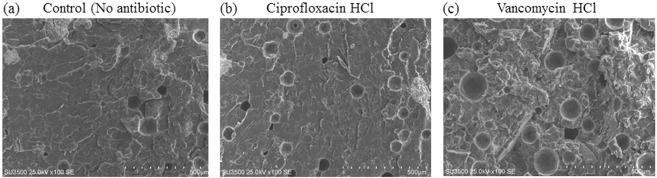
The microstructures of the fractured surfaces of dry bone cement, (**a**) cement without antibiotic (control group), (**b**) cement containing ciprofloxacin and (**c**) cement containing vancomycin.

**Figure 5:**
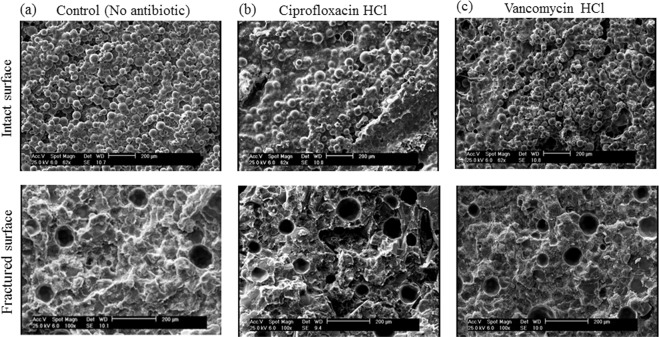
The microstructures of the intact and fractured surfaces of bone cement after 14 days of degradation at 37 ± 1 °C in distilled water, (**a**) cement without antibiotic (control group), (**b**) cement containing ciprofloxacin and (**c**) cement containing vancomycin.

The porosity of dry and wet control bone cement had a value of 2.02 and 2.08%, respectively. This value was increased to 3.05 and 3.67% by the addition of ciprofloxacin and vancomycin to the cement, respectively (Table 3, P < 0.001). Figure 4 shows the SEM images of dry bone cements and Fig. 5 presents images of cements’ surfaces after immersion in distilled water. ALBC developed a porous structure following 14 days of immersion with large pores of approximately 135 μm in average. It should be noted that the antibiotics concentrations of 2.5 and 5% had also similar effects (The images are not shown).

**Table 3:** Different properties (Mean (SD)) of porous bone cements containing 10% antibiotics and control group.

Group	Porosity (%)	ρ (g.cm^−3^)	Average of pore size (μm)	Total cumulative volume (mm^3^/g)
Dry sample	2.02 (0.005)	1.19	131.61 (2.43)	17.76 (0.76)
Wet sample	2.08 (0.07)	1.12	132.71 (3.24)	17.88 (0.99)
Cement + ciprofloxacin	3.05 (0.06)***	1.54	134.12 (2.3)	19.83 (0.96)
Cement + vancomycin	3.67 (0.15)***	1.59	136.50 (2.8)	23.05 (1.25)**

Dry sample and wet sample represent control group after storage in room temperature (23 ± 1 °C) and distilled water (37 ± 1 °C), respectively.

*In comparison to the wet sample (ANOVA, *p < 0.05, **p < 0.01, ***p < 0.001).

## Discussion

The most common important problems in joint replacement therapy is deep wound infection which can be reduced by adding antibiotic to the cement^25^. Gentamicin, teicoplanin, tobramycin, ciprofloxacin and vancomycin are common antibiotics used in orthopedic surgeries^12^. Different physicochemical properties of antibiotic powder including particle size and shape can also affect the mechanical properties of cements. Several studies that outline the characteristics of additive are available^26–28^.

In the present study for the first time a low molecular weight antibiotic (ciprofloxacin, MW = 367 g/mol) and a high molecular weight antibiotic (vancomycin, MW = 1485 g/mol) were added to the bone cement and their effect on bone cement properties compared. The water solubility of two antibiotics are similar. Results of this study show that both drugs caused substantial reduction of the cement strength in compression and bending tests. It has been demonstrated that this effect raised from antibiotic agglomeration in ALBC^11,29^.

We also found that antibiotic concentration in bone cement influenced the mechanical strength of the cement. It has been reported that the addition of ciprofloxacin decreased the compressive strength of BonOs and Biomecanica cements and this effect depended on antibiotic concentrations^12,30^. Results of this study showed that the use of low concentrations (2.5%) of both antibiotics maintained mechanical properties of the cement at the standard level (70 MPa)^14^. It is necessary to mention that this amount can provide the required concentration to prevent the growth of most of the common bacteria involved in orthopedic infections^31^. However, there are still some differences among literatures and the recommended antibiotic amount that does not compromise cement properties is not clearly defined yet. Pelletier and colleagues^32^ reported that adding high doses of vancomycin reduced the Simplex P and VersaBond cement compressive strength (16 and 18%) to below the standard level. Another study by Cheng *et al*.^33^, showed no negative effects on the cement compressive strength after adding 5% vancomycin, but other studies^11,34^ in agreement with our results reported that vancomycin at concentrations up to 2.5% (w/w) had no significant effect on the cement compressive strength. Despite the difference in cement brands and test modes employed, there would be a common outcome that the addition of antibiotics can reduce the cement strength and this effect is dependent on type and properties of used antibiotics, as mentioned by Paz *et al*.^35^.

Comparison of the effect of ciprofloxacin and vancomycin showed that MW of antibiotics could significantly affect the mechanical properties of cement, at high concentration (10% w/w, P = 0.002). Dunne *et al*.^22^ used chitosan, an ultra-high molecular weight macromolecule, reported significant reduction in the compressive and bending strengths of cements containing chitosan, after period of 28 days (p = 0.003).

Results of the present study showed that void and porosity were made in both control and ALBC groups due to different reasons; air entrapment or monomer evaporation and exposure to aqueous medium, as mentioned by Anagnostakos *et al*.^3^. These pores can reduce stiffness of acrylic cement by initiating micro-cracks. This phenomenon is exacerbated by the addition of antibiotics as a stress riser^29,36^. Elution of antibiotics from cement can also cause adverse effects on cement strength^17,22^. In the present study, density value of the control cement (1.18 g.cm^−3^) also declined over time (1.12 g.cm^−3^) due to interference with the liquid medium, which is in accordance with the other study^24^.

Finally, ageing was also an important factor that could affect the cement mechanical behavior following the implantation in orthopedic surgery. After aging, cements showed substantial reductions in strength. Therefore, it is important to determinate the most appropriate time to study mechanical properties. It should be emphasized that, in addition to cement brands, categories and brands of drugs, mixing and testing methods^37–39^, performing mechanical tests at different times can be one of the reasons lead to variation of reports in different studies.

Our results indicated that ALBC had the greatest decrease in strength after the first two weeks. Other studies also showed maximum reduction of mechanical properties after adding different additives in the first 14 days^17,40,41^. Following that, the cement strength increased on day 21 and 28. As stated in the other studies, the presence of antibiotic increases the penetration of water or body fluid into the cement which could explain the reduction of mechanical properties at the first 14 days^42^. Then, cement polymerization retrieved and increased the cement strength. This phenomenon is known as late polymerization, post hardening, and post curing process^32,43^. Based on these findings, the second week seems to be the optimal time interval to study the effects of supplementing PMMA bone cement with antibiotics.

## Conclusion

In order to study different concentrations of ciprofloxacin HCl and vancomycin HCl on the mechanical properties of PMMA bone cement over time, biomechanical tests and solid-state characterization were performed. In conclusion, the upper limit for the use of antibiotics in FIX 1 radiopaque bone cement is 2.5%. Concentration, properties, and type of the antibiotic added to cement affect the cement mechanical properties. According to the time polymerization phenomenon, the second week is probably the optimal time to evaluate the mechanical properties of ALBC. Furthermore, exposure to the liquid environment, resulting in voids and porosity increase in the cement. Finally, the importance of antibiotics molecular weight can be considered at high concentrations. However, further clinical studies are still needed to investigate.

## Supplementary information

Supplementary information.
