# Supplementary material for: Evaluation of the Effect of Ciprofloxacin and Vancomycin on Mechanical Properties of PMMA Cement; a Preliminary Study on Molecular Weight
Source: Sci Rep. 2020 Mar 4;10:3981. doi: 10.1038/s41598-020-60970-y (PMC7055350; doi:10.1038/s41598-020-60970-y)
Supplement: Supplementary file 1 — Supplementary information. [file 41598_2020_60970_MOESM1_ESM.pdf]

Evaluation of the effect of ciprofloxacin and vancomycin on mechanical properties of PMMA cement; a preliminary study on molecular weight

M. Gandomkarzadeh[1], H. R. Moghimi[1], A. Mahboubi[1, 2*]

[1]Department of Pharmaceutics and Nanotechnology, School of Pharmacy, Shahid Beheshti University of Medical Sciences, Tehran, Iran

[2]Food Safety Research Center, Shahid Beheshti University of Medical Sciences, Tehran, Iran

[*]Corresponding Author: A. Mahboubi

Postal Address: Niayesh Highway, Valiasr Ave, Tehran 1991953381, Iran

Email: a.mahboubi@sbmu.ac.ir

Tel: + 98 21 88200068; Fax: + 98 21 88665317

ORCID: 0000-0002-5140-8159

Supplementary Table S1 Details of antibiotic- loaded cement preparation.

Tests and Parameters	Concentration of antibiotic	Amount of antibiotic (mg)	Amount of powder/liquid component (g)	Weight of prepared cement (g)
Bending	0%	0	2.5/0.90	3.2
	2.5%	62.5	2.437/0.88	
	5%	125	2.375/0.85	
	10%	250	2.25/0.81	
Compression	0%	0	0.33/ 0.12	0.42
	2.5%	8.3	0.32/0.115	
	5%	16.6	0.31/0.11	
	10%	33.3	0.3/0.10	

Supplementary Table S2 Compression and bending properties of PMMA bone cements containing different concentrations of ciprofloxacin hydrochloride after immersion in distilled water at 37 ± 1 °C over 28 days (Mean (SD), n=5)

**Properties and concentrations**	**Time (days)**				
	1	7	14	21	28
Compressive strength (MPa)					
0 (Control)	83.21 (1.04)	78.86 (3.10)	75.97 (3.78)	72.75 (4.75)	72.39 (2.67)
2.5%	79.04 (3.92)	72.10 (2.81)*	69.88 (1.32)**	69.59 (4.17)	69.91 (2.52)
5%	72.43 (2.61)***	70.37 (3.90)**	66.85 (2.33)***	63.13 (2.06)*	64.51 (3.58)*
10%	71.24 (5.09)***	62.35 (2.68)***	56.42 (2.56)***	59.72 (6.96)**	60.05 (5.33)***
Compressive modulus (GPa)					
0 (Control)	1.61 (0.06)	1.32 (0.11)	1.18 (0.05)	1.11 (0.10)	1.12 (0.10)
2.5%	1.20 (0.13)***	1.13 (0.07)*	1.12 (0.08)	1.05 (0.07)	1.01 (0.07)
5%	1.15 (0.05)***	1.03 (0.19)**	1.07 (0.04)	1.00 (0.08)	0.99 (0.08)
10%	1.15 (0.10)***	0.88 (0.07)***	0.83 (0.08)***	0.84 (0.03)***	0.85 (0.05)***
Flexural strength (MPa)					
0 (Control)	49.79 (4.47)	47.69 (0.73)	44.36 (4.40)	42.47 (2.42)	43.87 (2.67)
2.5%	43.45 (4.30)*	42.38 (1.20)**	38.46 (1.30)**	40.50 (1.41)	39.94 (1.28)**
5%	37.45 (2.01)***	37.27 (2.28)***	35.33 (2.17)***	34.81 (1.05)***	34.54 (1.16)***
10%	37.19 (1.54)***	28.83 (3.29)***	29.79 (1.17)***	27.61 (1.65)***	25.72 (2.48)***
Flexural modulus (GPa)					
0 (Control)	2.35 (0.04)	2.23 (25.04)	2.15 (0.08)	2.12 (0.11)	2.12 (0.03)
2.5%	2.06 (0.05)***	2.01 (0.09)***	2.01 (0.09)**	2.01 (0.08)	1.98 (0.11)
5%	2.04 (0.07)***	1.94 (0.04)***	1.78 (0.05)***	1.74 (0.13)***	1.90 (0.06)*
10%	2.04 (0.10)***	1.90 (0.07)***	1.69 (0.08)***	1.69 (0.10)***	1.83 (0.19)**

* Significantly different from control group for same time (ANOVA, *: $p < 0.05$, **: $p < 0.01$, ***: $p < 0.001$)

Supplementary Table S3 Compression and bending properties of PMMA bone cements containing different concentrations of vancomycin hydrochloride after immersion in distilled water at 37 ± 1 °C over 28 days (Mean (SD), n=5)

**Properties and concentrations**	**Time (days)**				
	1	7	14	21	28
Compressive strength (MPa)					
0 (Control)	83.21 (1.04)	78.86 (3.10)	75.97 (3.78)	72.75 (4.75)	72.39 (2.67)
2.5%	73.77 (2.07)**	70.92 (3.33)**	70.65 (2.24)**	69.55 (1.95)	69.80 (2.53)
5%	71.51 (5.00)***	67.60 (2.93)***	64.82 (2.03)***	66.71 (3.54)	66.08 (1.59)**
10%	73.88 (4.86)**	56.42 (2.18)***	49.05 (2.01)***	59.90 (4.55)***	57.74 (1.75)***
Compressive modulus (GPa)					
0 (Control)	1.61 (0.06)	1.32 (0.11)	1.18 (0.05)	1.11 ± 0.10	1.12 (0.10)
2.5%	1.30 (0.11)**	1.13 (0.09)*	1.10 (0.07)	1.03 (0.08)	1.07 (0.07)
5%	1.28 (0.13)***	1.09 (0.11)*	1.05 (0.10)	1.01 (0.08)	1.01 (0.09)
10%	1.21 (0.10)***	1.02 (0.13)**	0.91 (0.08)***	0.95 (0.09)*	0.94 (0.08)*
Flexural strength (MPa)					
0 (Control)	49.79 (4.47)	47.69 (0.73)	44.36 (4.40)	42.47 (2.42)	43.87 (2.67)
2.5%	44.03 (1.55)*	40.42 (3.46)**	38.30 (1.61)**	39.77 (3.80)	39.69 (3.15)
5%	47.33 (2.51)	36.98 (2.74)***	32.94 (2.49)***	33.50 (3.87)**	38.30 (2.16)**
10%	35.06 (3.84)***	26.65 (2.76)***	25.16 (1.72)***	26.39 (1.57)***	31.33 (2.48)***
Flexural modulus (GPa)					
0 (Control)	2.35 (0.04)	2.23 (0.02)	2.15 (0.08)	2.12 (0.11)	2.12 (0.03)
2.5%	2.21 (0.14)	2.06 (0.14)	2.04 (0.12)	1.90 (0.06)*	1.93 (0.09)**
5%	2.26 (0.08)	1.96 (0.10)**	1.77 (0.12)***	1.77 (0.17)**	2.04 (0.10)
10%	1.91 (0.13)***	1.69 (0.18)***	1.43 (0.10)***	1.53 (0.02)***	1.91 (0.04)**

* Significantly different from control group for same amount of time (ANOVA, *: $p < 0.05$, **: $p < 0.01$, ***: $p < 0.001$)

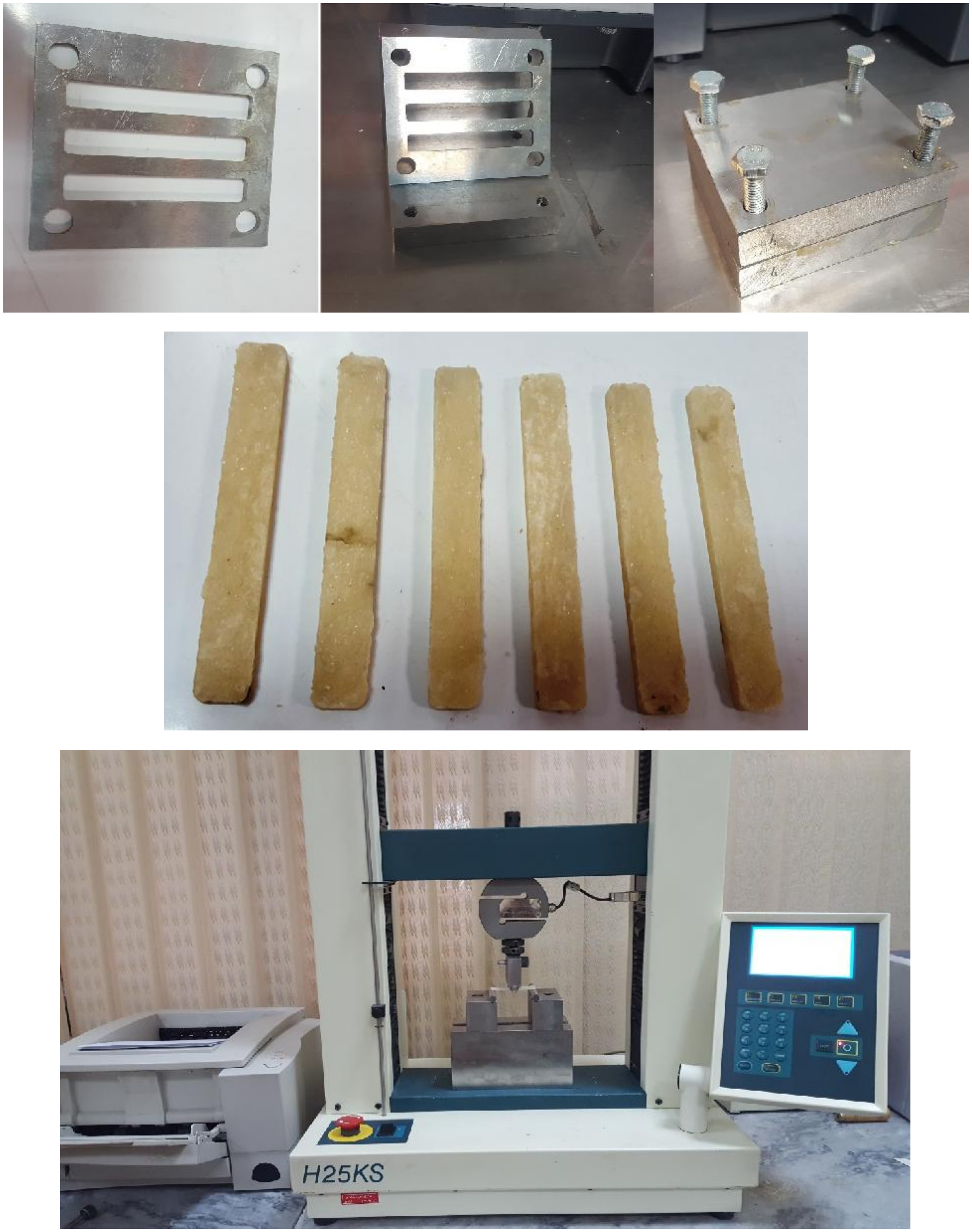

Supplementary Fig. S1 Image of used mold for preparation of rectangular strips (above), prepared cements (middle) and universal testing machine that was used for bending test (down).

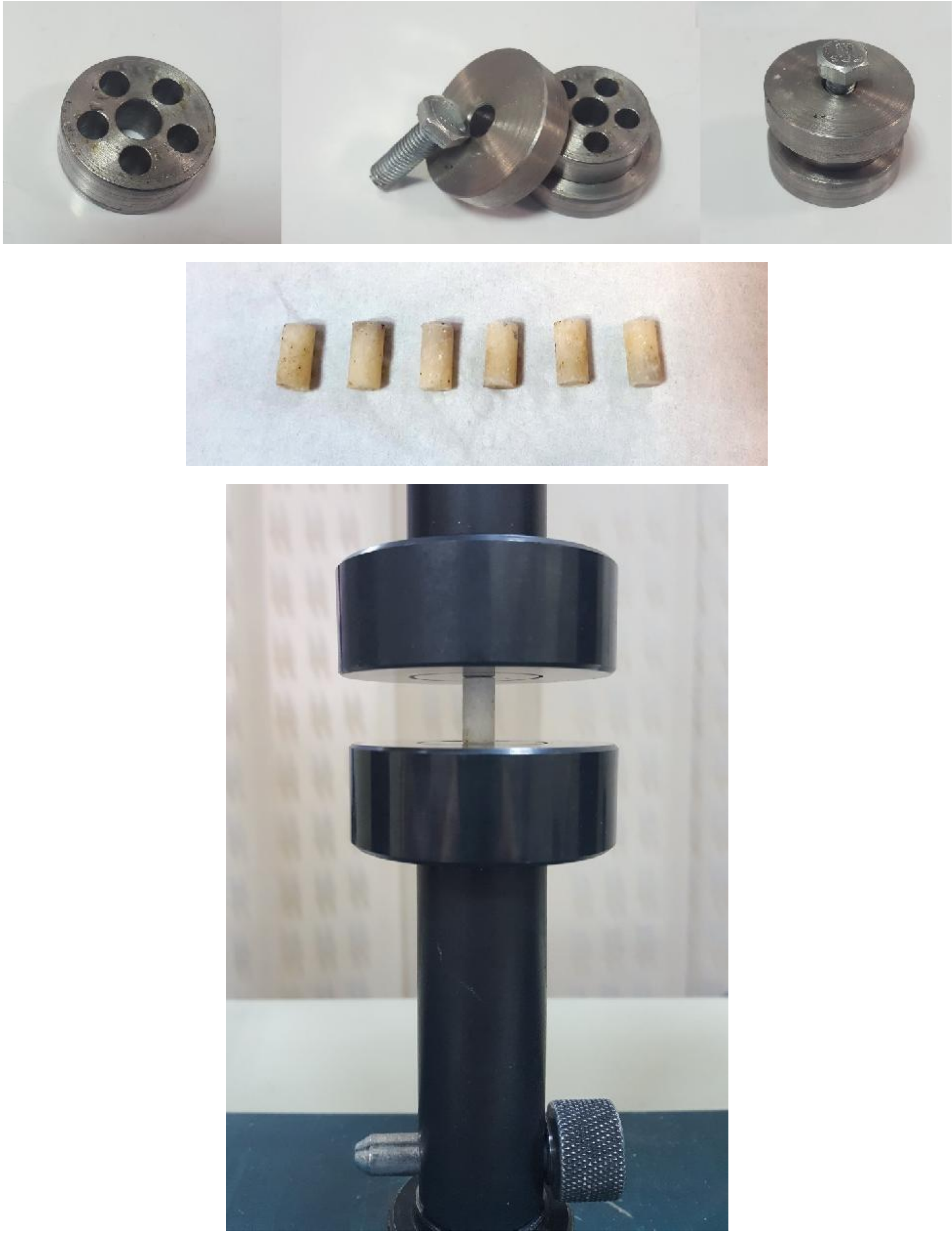

Supplementary Fig. S2 Image of used mold for preparation of small cylinder (above), prepared cements (middle) and a part of universal testing machine that was used for compression test (down).
